# Epidemiology of childhood overweight, obesity and their related factors in a sample of preschool children from Central Iran

**DOI:** 10.1186/s12887-019-1540-5

**Published:** 2019-05-20

**Authors:** Bahram Armoon, Mahmood Karimy

**Affiliations:** Social Determinants of Health Research Center, Saveh University of Medical Sciences, Tehran-Saveh freeway, Keveh Industrial Estate Company, Saveh, Postcode: 3914334911 Iran

**Keywords:** Birth weight, Perceived treat, Health responsibility, Body mass index

## Abstract

**Background:**

Childhood overweight and obesity are strongly associated with the psychological and physical health of those for the duration of the lifetime. The purpose of this study was to assess the epidemiology of childhood overweight and obesity and their related factors in Zarandieh city, of Iran, in 2017.

**Methods:**

In a cross-sectional study, 572 preschool-mother dyads from primary care Clinics were selected by multi-stage sampling method. BMI of the children and mothers were calculated using standard method and the demographic, children nutrition and physical activity habits; the mothers perceived threat toward obesity, and their life style data were collected by self-report questionnaires for the literate mothers and interviewing for illiterate mothers.

**Result:**

The prevalence of overweight and obesity in mothers was 30.8 and 20.3% respectively. This rate in children was 15.5 and 9.9% respectively. The multiple logistic regression analysis showed that variables of **mother’s** BMI, Birth weight, Mother’s employment, watching TV > 2 h/day, Computer games> 2 h/day and daily breakfast eating (≥4/week), perceived threat, health responsibility, stress management, physical activity, and healthy eating were the significant predictors of the child’s BMI respectively.

**Conclusions:**

Our results indicated that the prevalence of overweight and obesity are high in preschool children and their mothers. It seems that necessary to have suitable intervention programs to help mothers understand the serious risk of childhood obesity and the importance of creating a healthy lifestyle by them in childhood.

## Introduction

Childhood overweight (OW) and obesity (OB) is the global public health problem since it increases the risk of premature death, as well as developing Diabetes, cancer, heart diseases, and many other physical or social diseases and complications in adulthood [1]. It also causes undesirable psychological consequences, such as anxiety, depression, sleep disorders and low self-esteem, which affects the social and educational relationships of children [2, 3]. Researchers estimate that 79% of obese adolescents will become obese adults who are at increased risk of developing hypertension, and cardiovascular disease [4]. According to the WHO report, in 2016, over 340 million children and adolescents aged 5–19 years old were diagnosed as overweight/obese [5]. Researchers believe that the increased prevalence of obesity is the result of changes in the lifestyle of societies, such as the inactivity, collapse of energy balance, increased use of fast food and animal proteins, and increased use of technology [2, 6]. Several studies have shown that there is a relationship between lifestyle and health, and today, lifestyle changes are considered as an essential strategy for solving chronic health problems such as obesity [7, 8]. Most Asian countries, including Iran, are at a transition from traditional to western lifestyles. Lifestyle changes have increased the prevalence of overweight /obesity in these societies [9]. The study by Agha-Alinejad indicated the prevalence of overweight/obesity in Iranian preschool as 12% in boys and 22.5% in girls [10]. In 2017, non-communicable diseases accounted for 76% of total mortality in Iran [6]. The main risk factor for these diseases is the unhealthy lifestyle (e.g., getting extra calories, inactivity, and unhealthy nutrition). Healthy lifestyle plays a vital role in improving life expectancy and is associated with a reduction in the risk of death and recurrence of many diseases [11].

The construct of perceived threat is an individual’s perception about the seriousness of health problem [12], and their attitudes to one’s vulnerability to its problem [13, 14]. Previous studies demonstrated that perceived threat has a vital role in predictive to health behavior [13, 15]. Health professionals recognize children’s lifestyle as a function of parents’ behavior, belief and life styles [14]. Health professionals recognize children’s lifestyle as a function of parents’ behavior. Parents are considered to be the causes of the child’s BMI in the community, considering the rules for watching TV, Computer games, nutrition and using the car instead of walking [16, 17]. Children that have low levels of physical activity are more likely to watch TV than the others, and much-watching TV may cause to increase snacking and using up of high-fat, high-sugar, or high-calorie foods and the decrease of fruit and vegetable consumption and this lifestyle may cause to greater BMI among children [40]. Excessive use of computer and TV causes sedentary behavior in children, which is an essential determinant for developing OW/OB [8]. The role of mother for children is highlighted because the mother directly determines the social and physical environment of the child and indirectly affects their attitude, habits, and behaviors [14, 18]. The mother is the closest person to the child and the first person to shape their behavior and lifestyle. In multigenerational families that grandmothers have roles in preparing food for family members, even if mothers are employed the eating patterns have no significant transitions. Besides, in these situations, young children may have insufficient access to dense energy food. In the contexts that the mean of BMI Z-score is negative, the income should be dedicated to goods that are currently associated with healthy weight gain in LMIC. Because of the rapid growth in children aged 0–5 years, they require a high amount of energy. Following this, the growth of household food charges may cause only modest alterations in energy balance [34].

Meanwhile, the mother decides on the type and amount of household food [19]. Therefore, the mother plays an essential role in determining the weight of the child. Because people’s perception of health threats leads them to health behaviors, the present study is aimed to assess the effects of the perceived threat from obesity and mothers’ lifestyles on children’s BMI.

## Subjects and methods

### Design and sample

The present study is a descriptive, analytical cross-sectional study and the research community includes 576 mothers with 6-7 year-old children having medical records in the primary care clinics of Zarandieh city, placed in the Markazi Province of Iran, in 2017. After obtaining permission from the authorities of Saveh University of Medical Sciences and providing a list of all primary care clinics in the city, the research samples were selected through multi-stage sampling and entered into the study. The sampling method was as follows: in the first stage, all primary care clinics of the city including eight bases were selected by the census method. In the next stage, 72 children (36boys and 36 girls) were randomly selected from each center based on the family file number available in each base and entered the study with their mothers. In the third stage, the chosen mothers were called using the telephone number in the family file, and they were invited to the primary care clinics. The goals of the study were explained to the mothers, and self-administered questionnaires were completed by them in a separate room. After collecting the questionnaires, four questionnaires were deleted due to incomplete information, and the final analysis was done on 572 questionnaires. The inclusion criteria included mothers with records in primary care clinics and have children aged 6–7 years; exclusion criteria were the absence of informed agreement in the study. The heights and weight of the selected children and mothers were measured, and their BMIs were calculated. Height without shoes was measured using height gauge (SECA model) with a precision of 0.5 cm, and body weight was measured with clothes and without shoes with a digital scale of 500 g sensitivity, and finally, the BMI was calculated. For children, the obesity criterion was BMI ≥ 95, the overweight criterion was BMI between 85 and 95 percentiles, and normal weigh criterion was BMI between 50 and 85 percentiles [20]. For mothers, the overweight criterion was BMI between 25–29.99, and the obesity criterion was BMI ≥ 30 [16]. The reliability of the scale and meter used were confirmed using the test-retest method in three stages.

### Measures

Data were collected using written self-report questionnaires for the literate (middle/High school/university) and through interviews with the help of trained instructors for the low-literate (Illiterate/ Elementary). To design the questionnaire, the sample questionnaires used in similar studies were used [9, 13, 14, 18, 21]. The questionnaire included the following sections: 1. Demographics; this section provided descriptive information about the research samples such as mother’s age, child’s age, child’s birth weight, mother’s education, and mother’s occupation. 2. Perceived threat; this Scale is a 6 item tool that assesses the perceived threat of mothers towards childhood obesity (i.e., I am worried about the additional weight of my child). In this section, mothers’ answers were set based on the Likert 5-point scale ranging from 1 “ strongly disagree” to 5 “ strongly agree.” Therefore, the acquired score in this structure ranged between 6 and 30. Cronbach’s Alpha reliability of the Perceived threat was 0.82. A panel of 12 experts helped to evaluate the validity of this section; the technique of content validity ratio (CVR) and content validity index (CVI) was used. CVR value > 0.56 and a CVI value > 0.79 were considered “satisfactory.” In the next step, face validity was evaluated. For this purpose, 20 mothers who would not join later in the research was requested to score the importance of each question on the 5-point Likert scale from ‘1’ (not important at all) to ‘5’ (completely important). Only the questions with impact scores of 1.5 or above were accepted.

3. Child’s behavior by mother report: The child’s behavior in the two areas of “Nutrition and Physical Activity” was assessed through 16 items. Mother was asked about child’s intake of dietary indicators (Food habits, fast foods, fruit, and vegetables), physical activity habits, Duration of TV watching/computer games and sleep during the past 30 days. The reliability of this section was obtained by Cronbach’s Alpha as 0.78. 4. Health Promoting Lifestyle Profile II; this instrument consists of 52 items and six subscales (nutrition9 items, physical activity 8 items, health responsibility 9 items, stress management 8 items, interpersonal relationships 9 items, and spiritual growth 9 items) that assesses the health-promoting behavior of mothers. In this section, the mothers’ answers were set on a Likert scale ranging from 1 (Never) to 4 (always). The Iranian version of this scale was reliable by Taheri et al. [22]. In our study, the Cronbach’s alpha was determined as 0.81. The validity of the questionnaire was performed using Content Validity Index (CVI) and Content Validity Rate (CVR) with the assistance of 10 experts in the field of health sciences, nutrition, and behavioral sciences, and it was confirmed with CVI 81% and CVR 75%.

### Data analysis

The data were analyzed using SPSS 18. One-way analysis of variance (ANOVA) and T-Test were used to determine significant differences between the mean score of perceived threat, behavior and Health Promoting Lifestyle structures in two or more independent groups of demographic variables.

Multiple Logistic regression analysis was used to determine the factors affecting childhood obesity. At first, the variables were entered into the Univariate analysis, and the variables that became significant associations with childhood obesity (*P* ≤ 0.05) in this test, were entered into a multiple logistic regression model. The child’s BMI that was entered into the regression model as a dependent variable was classified as binary variables with code 0 for “normal,” and code 1 for “overweight/obese.”

### Ethics

The ethics committee of Saveh University of Medical Science approved this study and permission to conduct the research was obtained from this committee. Moreover, written consent from was taken from the mothers. Also, the teach-back method and interview were used to get informed consent in mothers with low levels of literacy.

## Results

### Demographic variables and obesity

The mean age of children and mothers was 6.3 ± 1.1 years and 32.6 ± 4.7 years, respectively. The prevalence rate of overweight and obesity in mothers based on the BMI index was 30.8% (*n* = 176) and 20.3% (*n* = 116), respectively. This rate in boys’ was 8.9 and 6.1%, as well as in girls was 6.6 and 3.8% respectively. About 36% (*n* = 189) of the mothers had a high school educational level, while the levels of elementary school, middle school, and the university had the following places with 23.8%(*n* = 125), 23%(*n* = 122) and 16.9%(*n* = 89). About 26% of the mothers were working, while the rest were homemakers. 287 children were boys, and the rest were girls. A total of 80 obese/overweight children (54.7%), and 175 normal weight children (46%) had a birth weight of more than 3000 g. The average number of children in families was 2.1 ± 1.6. The multiple logistic regression analysis showed that demographic variables of mother BMI, Birth weight, Mother’s employment were the most significant predictors of the child’s BMI respectively (Tables 1, 3). In the survey of perceived treats towards childhood obesity, 66.7% of mothers of children with normal weight worried about hazardous of OW in their children. This rate was 28% in mothers of OW/OB children (Table 2).

**Table 1:** Distribution of Mean and Standard Deviation and the Body Mass Index Based (BMI) on Demographic Variables of children

	BMI (children)		
	Normal (*n* = 379)	OW & OB (*n* = 146)	
Variables (mothers)	N (%)	N (%)	*P* Value
Age			
≤ 29	64 (16.8)	31 (21.2)	
30–39	206 (54.3)	68 (46.5)	
≥ 40	109 (28.7)	47 (32)	
education			
Illiterate/Elementary	70 (18.4)	55 (37.6)	
Middle school	88 (23.2)	34 (23.2)	
High school/university	221 (58.3)	57 (39)	
Socioeconomic status			
High	65 (17.1)	43 (29.4)	
Moderate	269 (70.9)	76 (52)	
Low	48 (12.6)	27 (18.4)	
BMI			
Underweight	14 (3.6)	6 (4)	
Normal	162 (42.7)	51 (34.9)	
OW	120 (31.6)	56 (38.3)	
OB	83 (21.8)	33 (22.6)

**Table 2:** Perceived treats towards childhood obesity among mothers

Item	Normal weight379						Overweight/obesity146					
	Strongly disagree/Disagree		No idea		strongly agree/agree		strongly disagree/disagree		No idea		strongly agree/agree	
	N	%	N	%	N	%	N	%	N	%	N	%
I am worry about excessive weight of my child	71	18.7	162	42.7	146	38.5	31	21	65	44.5	50	34.2
Loss of extra weight of my baby is one of my mental trouble	95	25	206	54	78	20.5	40	27.3	73	50	33	22.6
After buying fast-food meals for my child I fell sin and guilty	68	17.9	107	28.2	204	53.8	37	25.3	68	46.5	41	28
I believe that obese children are at the risk of some illnesses such as cardiovascular disease.	37	9.7	85	22.4	257	67.8	24	16.4	58	39.7	64	43.8
I believe that overweight /obesity of my child reduce my mental health and self-esteem	49	12.9	81	29	249	65.6	25	15.2	64	43.8	57	39
I believe that overweight/obesity hazardous to my children.	46	12.1	80	21	253	66.7	34	23.2	71	48.6	41	28

### The perceived threat of mothers and child behavior

The results revealed that the perceived threat was a significant variable in predicting of child’s BMI. Also, the mean score of perceived threat for mothers having children with normal BMI was higher than that for mothers having children with OB/OW, and the difference was statistically significant (*p* < 0.05). Also, the correlation coefficient indicated that behavior has a moderate positive and significant correlation with a perceived threat (*r* = 0.41, *p* < 0.001). In the “behavior” section, 58.6% of the mothers did not know how to calculate the BMI. 65 and 52% of the mothers set no time limit for their children’s watching TV or playing Computer games respectively. 61 and 54% of the children watched TV and played games for more than 2 h a day respectively. In the behavior section, Watching TV > 2 h/day, Computer games> 2 h/day and daily breakfast eating (≥4/week) were significant elements in predicting children’s BMI.

In the “Health Promoting Lifestyle” section, the mean scores of all the structures for mothers having children with normal BMI were significantly higher than mothers having children with OB/OW. The structures of health responsibility, stress management, physical activity, and healthy eating were the most important of significant predictors of child obesity respectively (*p* < 0.05) (Table 3).

**Table 3:** The univariate and multiple logistic regression analysis of demographic, behavioral and psychological variables for factors related to childhood overweight and obesity

	OW& OB (*n* = 146)	Normal weight (*n* = 379)	OR (95% CI)^a^	*P*	OR (95% CI)^b^	*P*
Demographics Variables	N (%)	N (%)				
Mother’s obesity						
No	57 (39)	176 (46)	1.0			
Yes	89 (61)	203 (54)	3.74 (2.12–7.20)	< 0.001	3.91 (1.35–6.86)	0.01
Birth weight						
< 3	66 (45)	204 (54.3)	1.0		1.0	
≥ 3	80 (55)	175 (45.7)	2.41 (1.26–4.98)	0.001	2.80 (1.24–5. 17)	0.02
Mother’s occupation						
Housewife	68 (46.6)	198 (52.3)	1.0		1.0	
Working	78 (53.4)	181 (47.7)	2.16 (1.04–3.35)	< 0.004	2.37 (1.18–4.21)	0.01
Behavioral Variables						
Watching TV > 2 h/day						
No	57 (39)	171 (45)	1.0		1.0	
Yes	89 (61)	208 (55)	3.39 (1.13–5.47)	< 0.001	3.51 (1.20–8.66)	0.01
Computer games> 2 h/day						
No	67 (46)	310 (81.8)	1		1	
Yes	79 (54)	69 (18.2)	3.06 (1.08–6.32)	0.001	3.40 (1.24–7.32)	0.01
Daily breakfast eating (≥4/week)						
Yes	56 (32)	299 (78.8)	1		1	
No	99 (68)	80 (21.2)	2.10 (1.04–4.16)	0.001	2.88 (1.19–6.67)	0.03
Psychological variables						
Perceived threat (mean, SD)	16.22 ± 4.35	21.56 ± 5.47	0.79 (0.72–0.84)	0.01	0.90 (0.85–0.94)	0.01
Health Promoting Lifestyle constructs						
Healthy eating	12.38 ± 2.24	18.76 ± 2.21	0.76 (0.61–0.90)	0.001	0.83 (0.75–0.89)	0.001
Health responsibility	19.95 ± 4.32	29.88 ± 3.79	0.71 (0.52–0.82)	0.03	0.80 (0.72–0.93)	0.05
Physical activity	8.47 ± 2.11	13.76 ± 2.02	0.68 (0.49–0.90)	0.01	0.77 (0.65–0.82)	0.001
Stress management	12.24 ± 3.15	16.87 ± 3.68	0.58 (0.37–0.82)	0.001	0.69 (0.44–0.79)	0.001

^a^Obtained from univariate analysis

^b^Adjusted OR obtained from multiple logistic regression analysis

## Factors associated with childhood overweight and obesity

Our result showed that there was about fourfold increase overweight children in mothers with BMI ≥25, (AOR = 3.91, 95% CI: 1.35, 6.86). Also, working mothers had two times likely to have overweight offspring’s (AOR = 2.37, 95% CI: 1.18, 4.21). The likelihood that birth weight ≥ 3000 g leads to overweight was two times (AOR = 3.91, 95% CI: 1.24, 5. 17).

Regarding children who spent their free time; by watching TV > 2 h/day (AOR = 3.51, 95% CI: 1.20, 8.66), and playing computer> 2 h/day (AOR = 3.4, 95% CI: 1.24, 7.32) were almost three times more likely to be overweight. Children who did not eat daily breakfast (≥4/week) were almost three times more likely to be overweight (AOR = 2.88, 95% CI: 1.19, 86.67).

Regarding to psychological factor and health promoting lifestyle constructs; perceived threat (AOR = 0.90, 95% CI: 0.85, 0.94), healthy eating (AOR = 0.83, 95% CI: 0.75, 0.89), health responsibility (AOR = 0.80, 95% CI: 0.72, 0.93), physical activity (AOR = 0.77, 95% CI: 0.65, 0.82) and stress management (AOR = 0.69, 95% CI: 0.44, 0.79) were almost less likely to be overweight (Table 3).

## Discussion

We can take a significant step towards preventing obesity by studying and analyzing the causes and predictors of obesity. In the present study, the mother OB/OW is an important variable affecting childhood obesity since children having mothers with obesity are more likely to be affected by obesity. The observed relationship between the mother’s weight and the child’s weight in the present study was similar to the results from other studies [2, 23]. For instance, in Bider-Canfield et al. study, the mother’s obesity increased the risk of the child’s obesity by 2.34 times [24]. In a similar survey of Danielzik et al. on 5–7-year-old German children, parent’s obesity was as the most important predictor of childhood obesity [2]. In consistent with our result, the effect of the mother’s BMI on the child’s BMI depends on both the genetics and the process of learning the mother’s behaviors and unhealthy lifestyles of children [3]. In recent decades, there is an increase in adults BMI because of changes in nutritional style. These changes are due to taking the high amount of saturated fats, sugar and refined foods (for example Fast-food) and low fiber in the daily diet, in addition to the decline in the daily physical activity [25, 26].

Our results indicate that the birth weight was a significant variable for predicting the preschool child’s BMI. The relationship between high birth weight and the increased risk of childhood obesity is also proven in the study of He et al. in China [27]. The similar finding has been reported by Gulliford et al. study in Trinidad and Tobago [28]. The relationship between high birth weight and the increased risk of childhood obesity is attributed to metabolic and endocrine activities or autonomic pathways. Furthermore, high birth weight remains a risk for obesity in children [29–32]. Based on meta-analysis research, high birth weight (> 4000 g) in comparison with normal birth weights (2500–4000 g), there is a high probability of childhood overweight (OR 1.66; 95% CI: 1.55–1.77). Accordingly, increased birth weight is associated with increased overweight risk, later on, proposing prenatal overfeeding as a key risk factor that leads to long-term obesity susceptibility [31]. Similarly, Qiao et al. [29], found that the full range of birth weights and the association of it with childhood obesity risk demonstrated that the birth weight > 3000 g increases the OR of overweight plus obesity during childhood. Consistent with previous studies [23, 33], in our study, a significant relationship was diagnosed between the child’s BMI and the mother’s employment. This means that the family income is likely to increase, which leads to increased purchasing power as well as increased diversity in purchasing food products. Moreover, the mother’s employment leads to the change in the child’s dietary pattern and increases the use of ready-made meals and high-calorie snacks, which consequently leads to an increased risk of developing obesity. Therefore, to illustrate the null findings several studies have specifications [34].

Our study showed mother’s perceived threat towards obesity was a significant factor in predicting for child’s BMI. The study of Azizi et al. [35] in tuberculosis patients revealed that effective threat perceptions are related to health decision-making. Similarly, Moore et al. [13] indicated that perceived threat could create motivation for losing weight and having more physical activity. In another study by Kim, found that that perceived threat is a crucial structure for motivating to prevention and improve the behaviors related to obesity in boys [36]. A recent meta-analysis of interventional studies highlights the role of perceived threat to facilitate behavior change [37]. It seems perceived threat is a critical determinant in adopting healthy behaviors since people react well to healthy messages only when they believe they are susceptible to risk such as obesity.

In this study, the period spent on watching TV and Computer game was a significant factor in predicting of child’s BMI. This finding is in line with the study of Brug’s et al. among school children in Europe [38], also, study of Hajian and Heidari [9] among preschool children in Iran, both of which showed that there was a positive and significant relationship between overweight and TV viewing and playing Computer games. Similar result have been reported in Katzmarzyk et al. [8], study in 9–11-year-old children from 12 countries, found that there was a significant relationship between childhood obesity and high TV viewing. [8]. Moreover, A study by Kelly et al. [39], indicated that children were exposed and influenced to high rates of TV advertising about unhealthy nutritional behaviors, which that can facilitate the consumption of unhealthy foods in children such as increased use of chips and cheese puffs [40].

As regards Health Promoting lifestyle variables, researchers have found significant relations between dietary habits and obesity. Similarly, our result indicated that dietary habits such as daily breakfast eating were a significant factor in predicting OW/OB. In line with this finding, a study by Vanhala on Finnish children proved that skipping breakfast is an important risk factor for developing childhood obesity [41]. The relationship between regular breakfast eating and fit weight for children could be justified by the fact that by regular eating of this main meal, the child’s appetite is full and they refuse to eat fatty snacks and junk food like chips and puffs. We also recognized that breakfast skipping and overweight/ obesity have a weak association with each other. Based on this finding the relationship between breakfast skipping, eating pathology, and obesity is not simple. For example, it is possible that eating pathology intervene in the relationship between breakfast skipping and overweight [42].

Previous studies have shown that Health responsibility is an important factor in promoting people’s health [43, 44]. In the present study, likewise, mothers who showed greater responsibility towards health were more likely to have normal weight children. Indeed, it seems that people who do not hold themselves responsible for their health and believe in the effect of fate, chance, and other factors on the development of diseases or health, do not try to correct their families’ unhealthy lifestyle, which consequently leads to the increased risk of developing diseases and complications like OW/OB in their families.

In this study, stress management was a significant factor in predicting of child’s BMI. Psychologists believe stress-induced overeating could be a contributing factor for obesity. In other words, when someone is stressed, they usually automatically and unconsciously look for ways to relieve their stress; the most common behavior of these people is eating, and children can learn this behavior from their mothers since parents are the children’s first role models. The study of Ng and Jeffery showed that there was a positive relationship between stress and fatty diets [45]. In a study Harding et al. [46], observed that people who are exposed to stress, are more likely to develop obesity. Also, Rydon et al. [47]. Showed that women with obesity were significantly more stressed.

Some studies reported that parent unhealthy lifestyles such as physically inactive, unhealthy eating practice are among the causes of childhood obesity [14, 18]. Similarly, in our study, mothers’ low physical activity could significantly predict the probability of developing childhood obesity. This is in line with the study of Rutledge et al. [48], found preschoolers’ weights were related to parent lifestyles. In another study, Etelson et al. [49], reported that parents lifestyle influence children in shaping dietary and physical activity habits. Also, an unhealthy lifestyle might increase the risk of childhood obesity. A study by Davis in Kansas City, USA showed that the parents’ healthy diet and physical activity has a vital role in creating the child’s ideal weight [50]. This finding should be taken seriously, since reduced physical activity leads to serious consequences, such as cardiovascular diseases, cancer, hypertension, diabetes, overweight, and obesity for the public health of people around the world, and it requires special attention of health workers to change the lifestyle of individuals and encourage to sports and physical activity. Indeed, this finding shows the importance of a healthy lifestyle in the parents. The present study had several limitations: Firstly, This data was collected only from the children’s mother. Secondly, the analysis of this study was based on cross-sectional data, thus does not enable to investigate the causal relationships.

## Conclusion

The results of the present study indicate that unhealthy behaviors and lifestyles are prevalent in children and their mothers. This problem could be a major risk to children’s weight loss programs in Iranian society. It seems necessary to have suitable intervention programs to help mothers understand the serious risk of childhood obesity and the importance of creating a healthy lifestyle by them in childhood. Accordingly, we recommend that encouraging active lifestyles and healthy diets should be considered as a public health priority. In addition, to improve our knowledge about genetic factors, educational and nutritional programs about obesity and related health outcomes should incept early in childhood that may prevent the increasing prevalence of childhood obesity and may reduce the frequency of obesity in children.

## Abbreviations

OB: Obesity
OW: Overweight
